# Role of hepatitis C virus core antigen assay in hepatitis C care in developing country

**DOI:** 10.1186/s43066-021-00146-z

**Published:** 2021-09-18

**Authors:** Ouafa Kallala, Saoussen Kacem, Imene Fodha, Bruno Pozzetto, Trabelsi Abdelhalim

**Affiliations:** 1 Research Laboratory for “Epidemiology and Immunogenetics of Viral Infections” (LR14SP02), Sahloul University Hospital, University of Sousse, Sousse, Tunisia; 2 Faculty of Pharmacy, University of Monastir, Avicenne Street, Monastir, Tunisia; 3 GIMAP EA3064, Faculté de Médecine, Université de Saint-Etienne/Université de Lyon, Lyon, France; 4 Laboratoire des Agents infectieux et d’Hygiène, CHU de Saint-Etienne, Saint-Priest-en-Jarez, France

**Keywords:** Core Antigen, Hepatitis C, Polymerase chain reaction

## Abstract

**Background:**

The World Health Organization (WHO) aims to achieve global hepatitis C elimination by 2030, defined as diagnosis of 90% of infected individuals and treating 80% of them. Current guidelines for the screening and diagnosis of hepatitis C infection denote using a relatively cheap screen with anti-hepatitis C virus (HCV) antibody immunoassay, followed by the much costlier molecular test for HCV RNA levels using polymerase chain reaction (PCR) assay to confirm active HCV infection. Simplification of the HCV evaluation algorithm to reduce the number of required tests could considerably expand the provision of HCV treatment especially in a developing country. This study investigates the performance of hepatitis C Core Antigen (HCV Ag) test by comparing HCV Ag results versus the results obtained with HCV ribonucleic acid (RNA) PCR which is considered the gold standard for the diagnosis of HCV infection.

**Results:**

Among the 109 anti-HCV positive sera, 96 were positive for both HCV Ag (> 3 fmol/L) and HCV RNA (> 15 IU/mL); 8 were negative for both tests, while the remaining 5 were positive for HCV RNA only. Considering the HCV RNA as gold standard; the sensitivity, specificity, positive predictive value (PPV) and negative predictive value (NPV) of HCV Ag test were found to be 95.05%, 100%, 100%, and 61.54%, respectively. HCV genotype was performed for 59 patients. The most common HCV genotype was genotype 1 (72.9%). Genotype 2 (15.3%) and genotype 3 (11.9%) were detected in the others samples. A high level of correlation was seen between HCV RNA and HCV Ag (*r* = 0.958, *p* < 0.001). The correlation for the samples that were genotyped 1 was significant (*r* = 0.966, *p* < 0.001).

**Conclusion:**

In our study, it was found that there was strong correlation between HCV RNA levels and HCV Ag levels. So, it can be used for a one-step HCV antigen test to diagnose active HCV infection.

## Background

Hepatitis C virus (HCV) infection is a global public health problem. Hepatitis C is found worldwide. The most affected regions are Eastern Mediterranean and European Regions, with the prevalence of 2.3% and 1.5%, respectively. Prevalence of HCV infection in other regions varies from 0.5 to 1.0%. Tunisia is a low endemicity country of HCV infection, with an average prevalence of 0.7% and a north-south gradient [1, 2].

HCV causes both acute and chronic infection. Acute HCV infection is usually asymptomatic, and is only very rarely (if ever) associated with life-threatening disease. About 15–45% of infected persons spontaneously clear the virus within 6 months of infection without any treatment. The remaining 55–85% of persons will develop chronic HCV infection. Of those with chronic HCV infection, the risk of cirrhosis of the liver is between 15 and 30% within 20 years [1].

Due to the fact that acute HCV infection is usually asymptomatic, few people are diagnosed during the acute phase. In those people who go on to develop chronic HCV infection, the infection is also often undiagnosed because the infection remains asymptomatic until decades after infection when symptoms develop secondary to serious liver damage [1, 3].

HCV infection is diagnosed in 2 steps. The first one is a screening for anti-HCV antibodies (anti-HCV) with a serological test which identifies people who have been infected with the virus. If the test is positive for anti-HCV, a nucleic acid test for HCV ribonucleic acid (RNA) is needed to determine active/previous infection state. Although no longer infected, they will still test positive for anti-HCV [1, 4, 5]. HCV RNA test should be performed in addition to anti-HCV in acute HCV infections during the window period and also in cases with certain immunodeficiencies [3, 4]. However, due to the expensiveness and high technicality of Polymerase chain reaction (PCR) machinery, it is impractical for low-income countries to routinely administer these tests [5, 6].

Simplification of the HCV evaluation algorithm to reduce the number of required tests could considerably expand the provision of HCV treatment. HCV Core Antigen (Ag) can be detected from 1 to 2 days following HCV RNA detection in serum and 33.2 (range 23–46) days prior to detection of HCV antibodies [7].

The aim of the present study is to evaluate the performance of HCV Ag test by comparing HCV Ag results versus the results obtained with HCV RNA polymerase chain reaction (PCR) which is considered the gold standard for the diagnosis of HCV infection.

## Methods

### Sample selection and characteristics

A total of 109 patient serum or plasma samples sent from different clinics to the Microbiology Laboratory of Sahloul University Hospital of Sousse, located in the Eastern Centre of Tunisia, for HCV RNA test were included in the study during the 2012–2015 period. Information on patients’ diagnoses, treatments, HCV activation markers, and microbiological laboratory data (anti-HCV, HCV RNA) were obtained from the hospital’s electronic information system and patient files.

### Detection of anti-HCV antibodies

Anti-HCV antibody test was performed by means of a chemiluminescent microparticle enzyme immunoassay (CMIA) (Cobas e411 Elecsys analyser, Roche, USA) in line with manufacturer’s recommendations. The “cut-off” value was considered 1.0 as per the kit procedure. It was considered as reactive which value of the test is ≥ 1.0.

### HCV RNA assay

Real-time PCR (COBAS AmpliPrep/COBAS Taq Man HCV real-time PCR, Roche Diagnostics, Germany) was used in line with manufacturer’s recommendations for HCV RNA quantitation in plasma samples. The assay has a limit of detection of 15 IU/mL with a linear quantitation window of 43–6.9 × 10^7^ IU/mL.

### HCV Ag assay

HCV Ag quantification were performed using Abbott ARCHITECT HCV Ag Assay (Abbott, Germany) with manufacturer’s recommendations. This is a two-step chemiluminescent microparticle immunoassay (CMIA) which is divided into a liquid phase with acridinium labeled murine anti-HCV antibodies, and a solid phase with paramagnetic microparticles. The manufacturer states the “cut-off” value as 3.00 fmol/L (0.06 pg/mL) and the quantitation upper limit as 20,000 fmol/L for this assay.

### Genotyping

#### HCV RNA extraction

The viral nucleic acid from HCV-infected patients’ plasmas was extracted using the QIAamp® DSP Virus Kit (QIAGEN) according to manufacturer’s instructions.

#### Reverse Transcription (RT) and Amplification of HCV 5′UTR/Core regions

This step was carried out using the HCV Amplification 2.0 Kit (LiPA) Simens®.

#### HCV genotyping

A Versant HCV genotype 2.0 assay (INNO-LiPA HCV 2.0) Simens® was performed according to the manufacturer’s instructions.

### Statistical analysis

Lineal regression analysis was used to assess the linear association between HCV Ag and HCV RNA concentrations as well as anti-HCV and HCV RNA concentrations in logarithmic scales.

Sensitivity, specificity, negative predictive value, positive predictive value, and accuracy values for the HCV Ag test were calculated versus the HCV RNA test (the gold standard). All data were analyzed by using SPSS software (SPSS; version 20.0). Statistical significance level was considered as 0.05.

## Results

Sixty-one (56.0%) of the 109 patients included in the study group were females and 48 (44.0 %) were males. Seventy eight (71.6%) were treatment-naive and 31 (28.4%) were under treatment.

HCV genotype was performed for 59 patients. Genotype 1 (43/59, 72.9%), genotype 2 (9/59, 15.3%), and genotype 3 (7, 11.9%) were detected in the study samples.

Among the 109 anti-HCV positive sera, 96 were positive for both HCV Ag (> 3 fmol/L) and HCV RNA (> 15 IU/mL); 8 were negative for both tests, while the remaining 5 were positive for HCV RNA only.

Considering the HCV RNA as gold standard, the sensitivity, specificity, positive predictive value (PPV), and negative predictive value (NPV) of HCV Ag test were found to be 95.05%, 100%, 100%, and 61.54% respectively (Table 1).

**Table 1:** Sensitivity and specificity of HCV Ag in predicted HCV RNA in 109 anti-HCV positive patients

		HCV RNA		
		Positive	Negative	Total
HCV Ag	Positive	96	0	96
	Negative	5	8	13
	Total	101	8	109

HCV Ag negative: HCV Ag < 3 fmol/L; HCV RNA negative: HCV RNA < 15 IU/mL

Sensitivity = 96/101 = 95.05%

Specificity = 8/8 = 100%

Positive predictive value (PPV) = 96/96 = 100%

Negative predictive value (NPV) = 8/13 = 61.54%

**Fig. 1:**
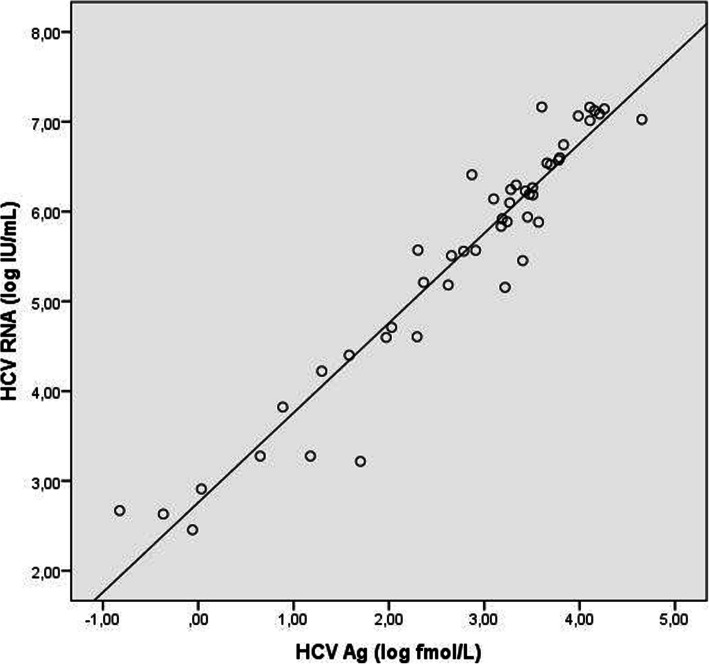
Correlation diagram of HCV Ag (log10 fmol/L) and HCV RNA (log10 IU/mL), Pearson’s correlation (*r* = 0.958, *p* < 0.001)

Figure 1 shows comparison between HCV RNA levels and HCV Ag levels using a logarithmic scale. Pearson’s correlation coefficient was 0.958 (*p* < 0.001) indicating good concordance, and the two tests were therefore considered as highly concordant.

**Fig. 2:**
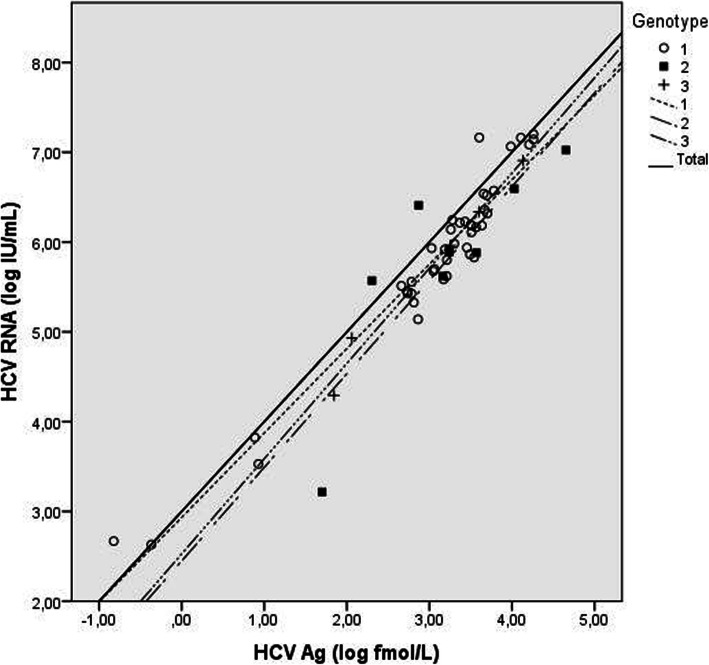
Correlation diagram of HCV Ag (log10 fmol/L) and HCV RNA (log10 IU/mL) according to genotype for 59 genotyped samples

The correlation for the samples that were genotyped 1 was significant (*p* < 0.001), given a Pearson coefficient value of 0.966. Figure 2 shows linear regressions for genotypes when plotted on log scales of HCV RNA and HCV Ag levels. Statistical correlation tests could not be performed regarding the genotypes other than genotype 1 due to the small number of non-genotype 1 samples detected in the present study.

Note that the lowest detection level of reactive HCV Ag (4.47 fmol/L) corresponded to 1890 IU/mL HCV RNA.

HCV Ag false negatives occurred at relatively low HCV RNA titers. Five samples (9.26%) positive for HCV RNA (38–813 IU/mL, mean value 406 IU/mL) were found to be negative for the HCV Ag test (0.00–1.08 fmol/L, mean value 0.506 fmol/L). Two of them were under treatment and three were treatment-naïve. HCV genotype could be determined for two samples among the five false negative results, and genotype 1 was detected in both cases.

## Discussion

Current guidelines for the screening and diagnosis of HCV denote using a relatively cheap screen with anti-HCV anti-body immunoassay, followed by the much costlier molecular test for HCV RNA levels using PCR assay to confirm active HCV infection. HCV Ag assays, which are easier to perform than real time-PCR also save time and are less expensive. Previous cost-effectiveness analysis showed that screening and evaluating HCV viremia using a strategy in which the two-step process is replaced by a one-step process could result in a net cost saving up to $44 per person screened [8, 9].

That is why, there has been a push for a one-step HCV antigen test to diagnose active HCV infection [10].

Detection of HCV Ag was initially described in late 90s. However, first generation antigen tests had soon become unpopular due to their lack of sensitivity [7]. The chemiluminescent immunoassay based new generation HCV Ag test used in this study has a sensitivity of ≤ 3 fmol/L, offering 16- to 25-fold sensitivity compared to preceding tests [11].

In our study, it was found that there was strong correlation between HCV RNA levels and HCV Ag levels. HCV Ag levels were detected, with a corresponding increase in HCV Ag levels to increased titer of viral RNA, with a high correlation coefficient of 0.958 (*p* < 0.001). Similar results were reported by various other studies [10–12]. Due to the excellent correlation between HCV Ag and HCV RNA concentrations, detection of HCV Ag in serum or plasma is useful as an indirect marker of HCV replication [13].

Sensitivity of the HCV Ag test, in the present study, was 95.05% compared to that of the RNA test. Previous studies which utilized the same HCV Ag test have reported a sensitivity of 75.8–99.5% for the HCV Ag test [11, 12, 14–16].

In our study, negative results were obtained with HCV Ag test for five samples with viral load of 38–813 IU/mL. In a study by Çetiner and colleagues [11], seven samples with positive HCV RNA results yielded negative HCV Ag results and viral load were lower than 10,000 IU/mL for these patients (six samples with viral loads of 17–178 IU/mL and one sample with a viral load of 2500 IU/mL). In another study by Ergünay and colleagues [15], the viral load was lower than 10^3^ IU/mL in samples which were negative for the HCV Ag test.

In the present study, specificity was found to be 100%. Ross and colleagues [17], Kesli and colleagues [12], Park and colleagues [18], Çetiner and colleagues [11] and Chang and colleagues [10] have also found a specificity level of 100%.

In the present study, PPV was 100% and there is no false positivity with the HCV Ag test. Same result was found by Çetiner and colleagues [11].

However, our negative predictive value (61.54%), similar to result find by Abdelrazik and colleagues [13], was lower than those of the other studies [12, 19]. This result was probably due to the small number of RNA negative samples, that is, 8 out of 109.

Consequently, all positive results found by the HCV Ag assay were also positive with the HCV RNA assay. However, all negative results found by the HCV core Ag assay were not negative with the HCV RNA assay. Thus, it can be concluded that a positive test with the HCV Ag (almost) always represents a true positive since it is highly specific. However, when there is a serum sample showing anti-HCV positivity, the negative results found by the HCV core Ag assay should be also confirmed by a HCV RNA assay [13].

There is increasing, albeit insufficient, evidence that genotype may affect antigen and RNA assay detection results to different extents [20]. In our study, genotype 1 was the predominant detected genotype, corresponding to the genotypic distribution of HCV in Tunisia [21–23]. Specially, there was a strong correlation between HCV RNA levels and HCV Ag levels in genotype 1 (*r* = 0.966). Similar result was found by Chang and colleagues (*r* = 0.945) [10]. In contrast, the correlation coefficient was considerably higher than those reported in previous study [24, 25]. This discordance may be explained by the variability of studied population and the use of different assays.

## Conclusion

The World Health Organization aims to achieve global hepatitis C elimination by 2030, defined as diagnosis of 90% of infected individuals and treatment initiation of 80% of eligible individuals. In the same directive line, Tunisia launched, in 2016, a national plan for the eradication of hepatitis C by 2023. The development and market approval of novel direct-acting antivirals (DAAs), such as sofosbuvir by itself or in combination with ledipasvir molecules currently available in Tunisia have dramatically changed the hepatitis C virus treatment landscape. Future studies are needed to evaluate the usefulness of HCV Ag quantification for the monitoring and detection of treatment failure, in patients treated with different DAAs.

## Data Availability

The data that support the finding of this study are available from the corresponding author upon reasonable request**.**

## Abbreviations

Anti-HCV: HCV antibodies
CMIA: Chemiluminescent microparticle enzyme immunoassay
DAAs: Direct-acting antivirals
HCV: Hepatitis C virus
HCV Ag: Hepatitis C core antigen
NPV: Negative predictive value
PCR: Polymerase chain reaction
PPV: Positive predictive value
RNA: Ribonucleic acid
WHO: World Health Organization
